# Identification of the optimal diagnostic criteria combination for reproductive tract diseases in dairy cows of 100 days in milk or more

**DOI:** 10.3168/jdsc.2024-0690

**Published:** 2025-03-03

**Authors:** J. Dubuc, J.C. Arango-Sabogal, V. Fauteux, J. Denis-Robichaud, S. Buczinski

**Affiliations:** 1 Faculté de médecine vétérinaire, Université de Montréal, Saint-Hyacinthe, Québec, J2S 7C6, Canada; 2 Independent researcher, Amqui, Québec, G5J 2N5, Canada

## Abstract

The objective of this study was to determine the optimal diagnostic criteria combination for purulent vaginal discharge (PVD) and endometritis (ENDO) in cows ≥100 DIM to predict the probability of pregnancy. A prospective observational study was conducted in 24 commercial Holstein herds selected by convenience. Cows ≥100 DIM identified as nonpregnant by veterinarians were systematically enrolled in the study and were examined with a Metricheck device (discharge) and a cytobrush device combined with a leukocyte esterase test. No reproductive tract treatments were allowed and cows were systematically enrolled on an ovulation synchronization protocol and inseminated. Data from 1,064 reproductive tract examination events from 918 different Holstein cows were included. Diagnostic criteria combinations of discharge score and esterase score were used to identify PVD and ENDO, respectively. Combinations were compared using mixed logistic regression models accounting for diagnostic criteria (PVD and ENDO), DIM, parity, and season, as well as herd and cow clustering as random effects. The optimal diagnostic criteria combination was chosen based on the variation explained when these thresholds were included in a model (lowest Akaike information criterion; AIC), and their ability to predict pregnancy status (highest area under the receiver operating characteristic curve; AUC). The lowest AIC (1,021) and highest AUC (0.898) were obtained with a combination of a discharge score ≥2 (flecks of pus or worse) for PVD and an esterase score ≥0.5 (trace of leukocytes or worse) for ENDO. Based on these criteria, the proportions of PVD and ENDO were 21.5% and 37.8%, respectively. The overall probability pregnancy at artificial insemination was 30.7%; it was higher in unaffected cows than in cows with reproductive tract diseases. In conclusion, the optimal diagnostic criteria combination for cows ≥100 DIM was a discharge score ≥2 for PVD and a esterase score ≥0.5 for ENDO, and cows with PVD or ENDO had poorer reproductive success than unaffected cows.

## Highlights

• Reproductive health affects the odds of pregnancy in cows .100 DIM.
• Combinations of thresholds for vaginal discharge and endometritis were assessed.
• Flecks of pus or worse and trace of leukocytes or worse was the best combination.

## Graphical Abstract

**Figure:**
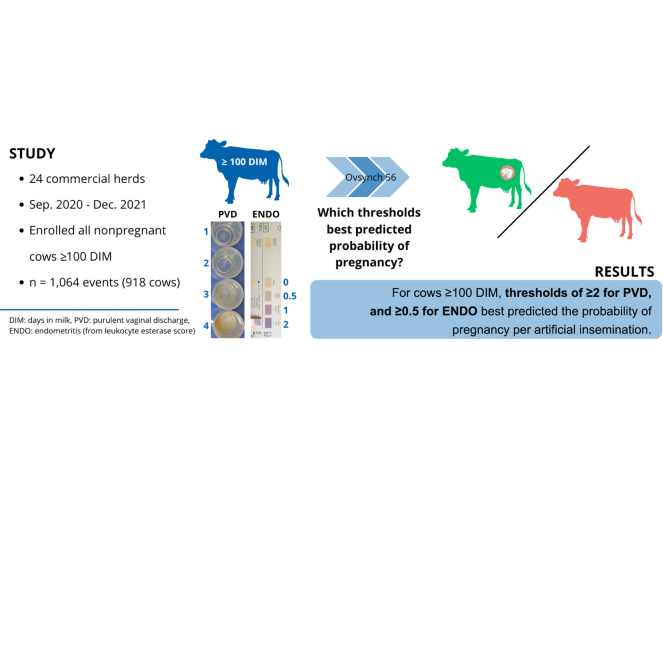
**Summary:** The objective of this study was to determine the optimal diagnostic criteria combination for purulent vaginal discharge (PVD) and endometritis (ENDO) in cows .100 days in milk (DIM) in order to predict the probability of pregnancy. During veterinary herd health visits, cows identified as nonpregnant were systematically examined to diagnose PVD (Metricheck device) and ENDO (leukocyte esterase test). Data from 1,064 reproductive tract examination events from 918 different cows (24 commercial herds) were analyzed in this prospective cohort study. The optimal diagnostic criteria combination was a vaginal score ≥2 (flecks of pus or worse) for PVD and a leukocyte esterase test score ≥0.5 (trace of leukocytes) for ENDO. The probability of pregnancy was lower in cows affected by PVD and ENDO than in unaffected cows.

Postpartum reproductive tract diseases such as purulent vaginal discharge (**PVD**) and endometritis (**ENDO**) have been extensively studied over the last 2 decades. They were shown to be frequent in postpartum dairy cows (Cheong et al., 2011; Dubuc and Denis-Robichaud, 2017) and to have detrimental effects on the subsequent reproductive performance of these cows (LeBlanc et al., 2002a; Dubuc et al., 2010; de Boer et al., 2015). Thus, surveillance of these 2 conditions on commercial dairy farms has become a frequent procedure during veterinary herd health visits conducted in commercial dairy farms in some areas of Québec, Canada. On-farm diagnosis of PVD can be performed using the Metricheck device (Simcro, Hamilton, New Zealand (McDougall et al., 2007), whereas ENDO diagnosis can be achieved using the cytobrush device combined with a leukocyte esterase test (Couto et al., 2013; Denis-Robichaud and Dubuc, 2015a; Arango-Sabogal et al., 2019). Although the accuracy of these easy-to-use tests is not perfect (Arango-Sabogal et al., 2019), they provide valuable information to farmers, veterinarians, and other advisors about the reproductive tract health status of cows during the postpartum period. As such, they can be used at the herd level to benchmark and identify herds at high risk of having poor success at their first insemination after calving (Dubuc and Denis-Robichaud, 2017).

Monitoring strategies for PVD and ENDO on commercial dairy farms focus on treating postpartum cows with intrauterine cephapirin (LeBlanc et al., 2002b; Kasimanickam et al., 2005; Denis-Robichaud and Dubuc, 2015b). However, many farmers do not implement such surveillance strategies on their farms, which leaves affected cows without treatment during the remaining of their lactation. Poorer reproductive performance throughout the lactation have been observed in cows with PVD and ENDO at 20 to 33 DIM when left untreated compared with treated ones (LeBlanc et al., 2002b; Kasimanickam et al., 2005), which suggest PVD and ENDO persist after the first month postpartum, likely even past 100 DIM. Moreover, ENDO on the day of artificial insemination (**AI**) was present in one out of 4 repeat breeder cows (3 or more AI; 209 ± 96 DIM), which was associated with decreased odds of pregnancy compared with no ENDO (Bogado Pascottini et al., 2018). There are, however, limited data available about the prevalence and impact of PVD and ENDO coming from systematic sampling in cows after their first insemination. Veterinarians deal on a daily basis with cows diagnosed nonpregnant to favor reproductive success of dairy herds. When the number of inseminations of a cow increases during her lactation, it could become strategic for veterinarians and farmers to identify if the cow is affected by PVD or ENDO to implement a treatment when necessary. Although implementing PVD and ENDO diagnostic procedures on nonpregnant cows after their first insemination would be feasible, it remains unclear what diagnostic criteria combination would be optimal for identifying reproductively compromised cows. Studies have demonstrated that the optimal diagnostic criteria for reproductive tract diseases can vary during the postpartum period. For instance, as the number of DIM increases, the diagnostic threshold of cytology to diagnose ENDO decreases (Dubuc et al., 2010). This change is also likely to apply to cows after their first insemination (e.g., ≥100 DIM), but no data are currently available to assess it. Therefore, our objective was to determine the optimal diagnostic criteria combination for PVD and ENDO in dairy cows ≥100 DIM to predict the probability of pregnancy per AI. We hypothesize that these criteria would reflect a presence of reproductive tract disease and would negatively impact the subsequent reproductive performance of affected cows compared with unaffected ones.

A prospective observational cohort study was conducted in 24 commercial Holstein dairy herds selected by convenience based on (1) being within a 1 h drive from the Bovine Ambulatory Clinic of the Université de Montréal (St-Hyacinthe, QC, Canada), (2) accepting to examine for PVD and ENDO all cows ≥100 DIM identified as nonpregnant after an insemination during veterinary herd health visits, and (3) implementing systematically an ovulation synchronization protocol on all these cows and following it rigorously. The study was approved by the Animal Care Committee of the Université de Montréal (20-Rech-2061). Each participating herd was visited every 14 d by a veterinarian and an animal health technician from September 2020 to December 2021. As part of the regular herd health visit, all cows bred ≥29 d were examined using transrectal palpation and B-mode ultrasonography. Cows identified as nonpregnant by the veterinarian were enrolled in the current study except for those cows assigned a “do not breed” status. It was possible for a cow to be enrolled multiple times in the study. The animal health technician performed the diagnostic procedures for vaginal discharge and esterase scores on all enrolled cows. First, the vulva was cleaned using paper towels. Then, a Metricheck device was inserted into the vagina to examine the presence of vaginal discharge which was scored as 0 = no discharge, 1 = clear mucus, 2 = mucus with flecks of pus, 3 = mucopurulent discharge, 4 = purulent discharge, and 5 = foul-smelling discharge (McDougall et al., 2007). After completion of Metricheck testing, a cytology sample from the endometrium was collected using the cytobrush technique (Kasimanickam et al., 2004) to perform a leukocyte esterase test. For that purpose, the cytobrush was immerged into a vial containing 1 mL of physiological saline (NaCl 0.9% Irrigation, Baxter Corporation, Mississauga, ON, Canada) and a leukocyte esterase strip (Multistix 10 SG, Bayer Corporation, Elkart, IN) was dipped in the solution (Denis-Robichaud and Dubuc, 2015a). A waiting period of 2 min was used before the colored esterase strip was read and scored as 0 = negative, 0.5 = trace of leukocytes, 1 = small amount of leukocytes, 2 = moderate amount of leukocytes, and 3 = large amount of leukocytes (Couto et al., 2013). Veterinarians and farmers were blinded to the discharge and esterase scores to avoid interference with the study protocol. Cows were not allowed to be treated with an intrauterine antimicrobial by veterinarians or farmers because it could influence the subsequent reproductive performance of the animals. All enrolled cows were assigned the same ovulation synchronization protocol (standard Ovsynch56; Brusveen et al., 2008) and were inseminated accordingly without any visual or activity-monitored heat detection. All cows were bred using AI. Following this insemination, enrolled cows followed the regular veterinary herd health visit examination procedure. If cows were eventually identified as pregnant from that insemination, it was considered to be a reproductive success. If cows were identified as nonpregnant from that insemination, it was considered to be a failure. Cows for which the study protocol was not followed or that were never rebred after enrollment were removed from data analysis.

A sample size of 1,070 enrolled cows was estimated based on having a difference of 10 percentage points in reproductive success between cows considered nonaffected by diseases (40%) and cows considered affected by diseases (30%), having an estimated prevalence of diseases of 35%, accounting for cow clustering (ρ = 0.05 and average cluster size = 2), and having a 95% confidence and 80% power (Dohoo et al., 2009).

Statistical analyses were computed using SAS version 9.4 (SAS Institute Inc., Cary, NC). The “reproductive tract examination event” was the unit of interest in this study. Additional data collected included parity, DIM, interval between the reproductive tract examination event and subsequent insemination, and culling information. Season of the reproductive tract examination event was also recorded as winter (January to March), spring (April to June), summer (July to September), and fall (October to December). Descriptive statistics were computed using the MEANS and FREQ procedures. Dummy variables were created for PVD and ENDO diagnostic criteria separately, using multiple thresholds for each condition (presented in Table 1). A total of 12 mixed logistic regression models (one model for each combination of diagnostic criteria) having the probability of pregnancy per AI (**P/AI**) as the dependent variable were computed (PROC GLIMMIX). In these models, cow and herd were included as random intercepts to account for the repeated measures and clustering structure at the cow and herd levels. Because of their potential confounding effect, season, DIM, and parity were included in all models. Since these models were non-nested, they were compared using the Akaike information criterion (**AIC**) values; models with better fit providing lower AIC values than poorer models (Burnham and Anderson, 2003; Dohoo et al., 2009). This approach allows to compare various diagnostic criteria combinations as each combination is tested as a predictor in a different non-nested model (Denis-Robichaud and Dubuc, 2015a). We also measure the discrimination capacity of each model by randomly splitting the dataset in training (0.70) and testing (0.30) sets, which were used to calculate the area under the receiver operating characteristic curve (**AUC**; Steyerberg et al., 2010). Using the combination with the lowest AIC (Burnham and Anderson, 2003), and the highest AUC, the proportion of cows having reproductive tract diseases was computed. The interaction between PVD and ENDO was included in the final model. The LSMEANS of P/AI stratified by disease status were computed using the optimal diagnostic criteria combination and tested using a Tukey-Kramer comparison.

**Table 1:** Number of unaffected cows, and cows with purulent vaginal discharge (PVD) or endometritis (ENDO) using various thresholds for the discharge and esterase scores, as well as the proportion of pregnant cows in each group^1^

Diagnostic criteria		Number^2^ of cows (% pregnant)			AIC	AUC
Discharge score	Esterase score	Unaffected	PVD	ENDO		
**≥2**	**≥ 0.5**	**528 (43.0)**	**229 (24.1)**	**402 (26.1)**	**1,021.1**	**0.898**
	≥1	712 (41.3)	229 (24.1)	191 (20.1)	1,028.4	0.851
	≥2	797 (40.2)	229 (24.1)	79 (15.2)	1,030.6	0.796
	≥3	839 (39.1)	229 (24.1)	22 (13.6)	1,034.1	0.717
≥3	≥0.5	652 (41.1)	75 (18.7)	402 (26.1)	1,026.8	0.844
	≥1	837 (40.0)	75 (18.7)	191 (20.1)	1,029.8	0.825
	≥2	929 (38.9)	75 (18.7)	79 (15.2)	1,034.1	0.768
	≥3	978 (37.7)	75 (18.7)	22 (13.6)	1,039.0	0.691
≥4	≥0.5	686 (41.0)	13 (7.7)	402 (26.1)	1,032.4	0.732
	≥1	880 (39.5)	13 (7.7)	191 (20.1)	1,036.7	0.675
	≥2	985 (38.0)	13 (7.7)	79 (15.2)	1,039.7	0.597
	≥3	1,035 (36.9)	13 (7.7)	22 (13.6)	1,048.9	0.566

1 Non-nested models predicting the probability of pregnancy per AI in 1,064 events from 918 different Holstein dairy cows ≥100 DIM from 24 herds were assessed using the AIC value. Training and testing sets (0.7:0.3) were then created, and the area under the receiver operating characteristic curve (AUC) of each model was calculated from the predictions (training set) applied to a testing set. The combination with bold font was selected for subsequent analyses because it had the lowest AIC and highest AUC.

2 Within a row, the sum of may be greater than 1,064 because cows can be positive for both PVD and ENDO simultaneously.

A total of 1,092 events from 934 cows were included in this study, but 28 were removed from data analysis because they did not follow rigorously the reinsemination protocol (n = 24) or were never reinseminated at all (n = 4). The removed events had a median (range) DIM and parity of 164 (104–427) and 2 (1–7), respectively. Their Metricheck score 0 (21.4%), 1 (60.7%), 2 (10.7%), 3 (3.6%), 4 (3.6%), and 5 (0%), and their esterase scores were 0 (67.9%), 0.5 (17.9%), 1 (3.6%), 2 (7.1%), and 3 (3.6%). In the end, data from 1,064 events from 918 cows from 24 herds were used for statistical analyses. Of these herds, 22 housed their cows in tiestall barns (2 were freestalls) and 18 fed their cows TMR (6 were component-fed). Median (range) values for DIM, parity, and the interval between the event and subsequent insemination were 168 (101–454), 2 (1–9), and 10 (10–10), respectively. The proportions of events by season were 21.1% (n = 225) for winter, 19.9% (n = 212) for spring, 22.0% (n = 234) for summer, and 36.9% (n = 393) for fall. Metricheck scores were 0 (20.1%; n = 214), 1 (58.4%; n = 621), 2 (14.5%; n = 154), 3 (5.8%; n = 62), 4 (1.2%; n = 13), and 5 (0%; n = 0). Esterase scores were 0 (62.2%; n = 662), 0.5 (19.8%; n = 211), 1 (10.5%; n = 112), 2 (5.4%; n = 57), and 3 (2.1%; n = 22). The overall P/AI was 36.4% (n = 387).

Table 1 presents all diagnostic criteria combinations, their AIC and AUC values, and the proportion of events associated with them in the study population. The optimal diagnostic criteria combination was a vaginal score ≥2 for PVD and a leukocyte esterase test score ≥0.5 for ENDO. Using these criteria, proportions of 21.5% (n = 229) and 37.8% (n = 402) of events were considered as PVD and ENDO, respectively. Looking at the combination of diseases, 12.6% (n = 134) were considered as PVD only, 28.9% (n = 307) were ENDO only, and 8.9% (n = 95) were considered as PVD and ENDO. At the herd level, the median (minimum–quartile 1–quartile 3–maximum) proportion of positive events for PVD was 18.6% (4.5%–9.1%–19.9%–37.4%) and 38.3% (9.1%–24.2%–45.3%–78.7%) for ENDO. The P/AI obtained from the optimal combination model are presented in Figure 1, showing that cows with a reproductive tract condition had lower P/AI than unaffected cows. The interaction between PVD and ENDO was not statistically significant (*P* = 0.56), but the numerical difference in P/AI for cows with both conditions (Figure 1; 13.5% ± 12.2%) compared with cows with PVD only (30.1% ± 7.5%) or ENDO only (32.4% ± 5.1%) suggests this should be explored in a study with a larger sample size.

**Figure 1:**
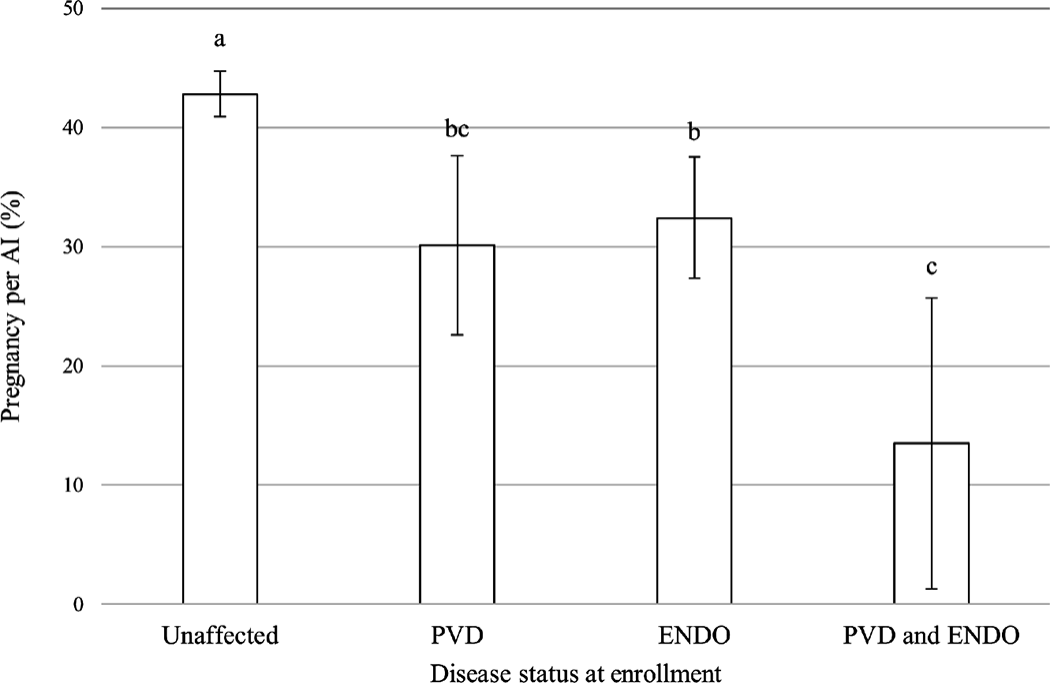
Least squares means (±SEM) of the probability of pregnancy per AI in 1,064 events from 918 Holstein cows ≥100 DIM. Values are stratified by reproductive tract disease status, showing that unaffected cows are more likely to become pregnant than cows with PVD, ENDO, or both PVD and ENDO. Different letters indicate a significant difference (*P* < 0.05; Tukey-Kramer). PVD = purulent vaginal discharge (vaginal discharge: mucus with flecks of pus or worse); ENDO = endometritis (esterase test: trace leukocytes or more).

The present study allowed to identify the optimal diagnostic criteria combination for PVD and ENDO to predict the P/AI in cows ≥100 DIM. Our results suggest that cows identified as nonpregnant during veterinary herd health visits and affected by reproductive tract diseases (PVD or ENDO) are more likely to have a poorer subsequent reproductive performance than unaffected cows. For instance, diagnostic criteria as subtle as a vaginal discharge with flecks of pus (Metricheck) or a trace of leukocytes (esterase) were sufficient to be associated with a decreased reproductive success at subsequent insemination. These diagnostic criteria are interesting for veterinarians who deal daily with cows bred multiple times and repeatedly identified as nonpregnant during herd health visits.

These thresholds were lower than what was found for the early postpartum period (Dubuc et al., 2010; Couto et al., 2013; Arango-Sabogal et al., 2019). This aligned with other studies where criteria were identified at different moments in the postpartum period, for which lower thresholds were identified as samples were collected further from calving. For example, diagnosing ENDO using the proportion of polymorphonuclear cells from cytological uterine samples, 18% was suggested for samples collected between 20 and 33 DIM, 4% for samples collected between 53 and 59 DIM, and even 1% samples collected at AI (with a cytotape; 122 ± 54 DIM; Kasimanickam et al., 2004; Dubuc et al., 2010; Bogado Pascottini et al., 2017). Reproductive tract diseases and inflammation are difficult to assess systematically in dairy cows past their voluntary waiting period in commercial contexts as cows are inseminated, and ideally pregnant, quickly. However, the modulation of inflammation has been shown to play a key role in reproductive tract diseases and their impact on subsequent reproduction (Velázquez et al., 2019; Pascottini and LeBlanc, 2020; LeBlanc, 2023). Briefly, inflammation of the reproductive tract has repeatedly been associated with inadequate uterine environment, and disrupted ovarian function, resulting in poor oocyte quality and poor reproductive performance. Our findings also show that inflammation in the reproductive tract after 100 DIM, even at low level, still has a negative impact on reproductive performance of dairy cows.

Our herd selection process was based on convenience for logistical reasons and this could have influenced our results. For instance, our reported proportion of cows affected by PVD and ENDO might not be reflective of the entire population of dairy cows and herds. It is possible that our herd population reflects a group of farmers that are highly motivated by achieving high reproductive success at the herd level and are willing to participate to such an intensive data collection. It is also possible that herds having reproductive problems in cows ≥100 DIM might be more inclined to participate to improve their cow and herd performance. In herds with high motivation, it is possible that the association would have been biased toward the null hypothesis as other strategies could have improved P/AI in cows with diseases. In herds with reproductive problems, for example due to management practices, the results could have been biased toward the null hypothesis if unaffected cows had low P/AI, or away from the null hypothesis if cows with reproductive diseases had worse P/AI. The magnitude of these biases was not assessed in the present study, and we think that the inclusion of multiple herds likely minimized their impact and allowed for generalizable conclusions.

One should also keep in mind that the accuracy of the Metricheck and the leukocyte esterase tests are not perfect to diagnose reproductive tract diseases (Arango-Sabogal et al., 2019). In this context, the optimal diagnostic criteria combination identified in our study were reported to have a sensitivity and specificity of 64.2% and 96.6% for PVD, and 84.0% and 53.6% for ENDO, respectively, in a population of postpartum cows (Arango-Sabogal et al., 2019). It remains unclear if such accuracy measures would be similar in cows ≥100 DIM. For instance, these values could change over time when cows experience an increasing number of estrus. The imperfect sensitivity and specificity of PVD and ENDO found previously highlight the importance of exploring new tools to better identify reproductive diseases. This deserves further investigation in future studies, but our study still identified the thresholds for these conditions with the best predictive ability for P/AI. Diagnosing PVD and ENDO in cows ≥100 DIM can be a useful tool for veterinarians to assess reproductive tract health when investigating poor reproductive performance in dairy cows after their first AI. This information could support the implementation of preventive measures, but would also identify cows that would benefit from a treatment (Dubuc et al., 2025).

In conclusion, the optimal diagnostic criteria combination we found in this study population for cows ≥100 DIM identified as nonpregnant at the veterinary herd health visits was a vaginal score ≥2 for PVD and a leukocyte esterase test score ≥0.5 from ENDO. This combination allowed to discriminate P/AI between cows affected or not by reproductive tract diseases.

## References

[bib1] Arango-Sabogal J.C., Dubuc J., Krug C., Denis-Robichaud J., Dufour S. (2019). Accuracy of leukocyte esterase test, endometrial cytology and vaginal discharge score for diagnosing postpartum reproductive tract health status in dairy cows at the moment of sampling, using a latent class model fit within a Bayesian framework. Prev. Vet. Med..

[bib2] Bogado Pascottini O., Hostens M., Opsomer G. (2018). Cytological endometritis diagnosed at artificial insemination in repeat breeder dairy cows. Reprod. Domest. Anim..

[bib3] Bogado Pascottini O., Hostens M., Sys P., Vercauteren P., Opsomer G. (2017). Cytological endometritis at artificial insemination in dairy cows: Prevalence and effect on pregnancy outcome. J. Dairy Sci..

[bib4] Brusveen D.J., Cunha A.P., Silva C.D., Cunha P.M., Sterry R.A., Silva E.P.B., Guenther J.N., Wiltbank M.C. (2008). Altering the time of the second gonadotropin-releasing hormone injection and artificial insemination (AI) during Ovsynch affects pregnancies per AI in lactating dairy cows. J. Dairy Sci..

[bib5] Burnham K.P., Anderson D.R. (2003).

[bib6] Cheong S.H., Nydam D.V., Galvão K.N., Crosier B.M., Gilbert R.O. (2011). Cow-level and herd-level risk factors for subclinical endometritis in lactating Holstein cows. J. Dairy Sci..

[bib7] Couto G.B., Vaillancourt D.H., Lefebvre R.C. (2013). Comparison of a leukocyte esterase test with endometrial cytology for diagnosis of subclinical endometritis in postpartum dairy cows. Theriogenology.

[bib8] de Boer M., Buddle B.M., Heuer C., Hussein H., Zheng T., LeBlanc S.J., McDougall S. (2015). Associations between intrauterine bacterial infection, reproductive tract inflammation, and reproductive performance in pasture-based dairy cows. Theriogenology.

[bib9] Denis-Robichaud J., Dubuc J. (2015). Determination of optimal diagnostic criteria for purulent vaginal discharge and cytological endometritis in dairy cows. J. Dairy Sci..

[bib10] Denis-Robichaud J., Dubuc J. (2015). Randomized clinical trial of intrauterine cephapirin infusion in dairy cows for the treatment of purulent vaginal discharge and cytological endometritis. J. Dairy Sci..

[bib11] Dohoo I.R., Martin S.W., Stryhn H. (2009).

[bib12] Dubuc J., Arango-Sabogal J.C., Fauteux V., Denis-Robichaud J., Buczinski S. (2025). Randomized controlled trial of intrauterine cephapirin treatment in cows of 100 days in milk or more affected by reproductive tract diseases. JDS Commun..

[bib13] Dubuc J., Denis-Robichaud J. (2017). A dairy herd-level study of postpartum diseases and their association with reproductive performance and culling. J. Dairy Sci..

[bib14] Dubuc J., Duffield T.F., Leslie K.E., Walton J.S., LeBlanc S.J. (2010). Definitions and diagnosis of postpartum endometritis in dairy cows. J. Dairy Sci..

[bib15] Kasimanickam R., Duffield T.F., Foster R.A., Gartley C.J., Leslie K.E., Walton J.S., Johnson W.H. (2004). Endometrial cytology and ultrasonography for the detection of subclinical endometritis in postpartum dairy cows. Theriogenology.

[bib16] Kasimanickam R., Duffield T.F., Foster R.A., Gartley C.J., Leslie K.E., Walton J.S., Johnson W.H. (2005). The effect of a single administration of cephapirin or cloprostenol on the reproductive performance of dairy cows with subclinical endometritis. Theriogenology.

[bib17] LeBlanc S.J. (2023). Review: Postpartum reproductive disease and fertility in dairy cows. animal.

[bib18] LeBlanc S.J., Duffield T.F., Leslie K.E., Bateman K.G., Keefe G.P., Walton J.S., Johnson W.H. (2002). Defining and diagnosing postpartum clinical endometritis and its impact on reproductive performance in dairy cows. J. Dairy Sci..

[bib19] LeBlanc S.J., Duffield T.F., Leslie K.E., Bateman K.G., Keefe G.P., Walton J.S., Johnson W.H. (2002). The effect of treatment of clinical endometritis on reproductive performance in dairy cows. J. Dairy Sci..

[bib20] McDougall S., Macaulay R., Compton C. (2007). Association between endometritis diagnosis using a novel intravaginal device and reproductive performance in dairy cattle. Anim. Reprod. Sci..

[bib21] Pascottini O.B., LeBlanc S.J. (2020). Modulation of immune function in the bovine uterus peripartum. Theriogenology.

[bib22] Steyerberg E.W., Vickers A.J., Cook N.R., Gerds T., Gonen M., Obuchowski N., Pencina M.J., Kattan M.W. (2010). Assessing the performance of prediction models: A framework for traditional and novel measures. Epidemiology.

[bib23] Velázquez M.M.L., Peralta M.B., Angeli E., Stassi A.F., Gareis N.C., Durante L., Cainelli S., Salvetti N.R., Rey F., Ortega H.H. (2019). Immune status during postpartum, peri-implantation and early pregnancy in cattle: An updated view. Anim. Reprod. Sci..

